# Predictive Effect of Systemic Immune-Inflammation Index Combined With Prognostic Nutrition Index Score on Efficacy and Prognosis of Neoadjuvant Intraperitoneal and Systemic Paclitaxel Combined With Apatinib Conversion Therapy in Gastric Cancer Patients With Positive Peritoneal Lavage Cytology: A Prospective Study

**DOI:** 10.3389/fonc.2021.791912

**Published:** 2022-01-19

**Authors:** Ping’an Ding, Peigang Yang, Chenyu Sun, Yuan Tian, Honghai Guo, Yang Liu, Yong Li, Qun Zhao

**Affiliations:** ^1^ The Third Department of Surgery, The Fourth Hospital of Hebei Medical University, Shijiazhuang, China; ^2^ Internal Medicine, AMITA Health Saint Joseph Hospital Chicago, Chicago, IL, United States

**Keywords:** apatinib, abdominal exfoliation cytology positive, gastric cancer, systemic immune-inflammation index (SII), prognostic nutrition index (PNI)

## Abstract

**Background:**

Gastric cancer with only peritoneal lavage cytology (GC-CY_1_) is a special type of gastric cancer, which is defined as stage IV. The pre-treatment systemic immune-inflammation index (SII) and prognostic nutritional index (PNI) are representative blood indexes of systemic inflammatory response and nutritional status. However, the clinical significance of combined detection of these two indexes is still unclear. This study aims to evaluate the clinical value of the new score system by combining SII and PNI (SII-PNI score) as a predictor of efficacy and prognosis after neoadjuvant intraperitoneal and systemic (NIPS) paclitaxel combined with Apatinib conversion therapy for GC-CY_1_ patients.

**Methods:**

We registered a prospective clinical study involving 36 GC-CY_1_ patients from April 2018 to August 2019 (NCT03718624). All patients underwent re-laparoscopic exploration after treatment. According to free cancer cells (FCCs) status, these patients were divided into FCCs group and non-FCCs group. The SII-PNI score ranged from 0 to 2 as follows: score of 2, high SII (≥512.1) and low PNI (≤52.9); score of 1, either high SII or low PNI; score of 0, no high SII nor low PNI.

**Results:**

All patients underwent re-laparoscopic exploration after 3 cycles of NIPS paclitaxel and Apatinib conversion therapy. Among them, 28 cases (77.78%) were in non-FCCs group, and 8 cases (22.22%) were in FCCs group. The SII-PNI score of non-FCCs patients was significantly lower than that of FCCs patients (p=0.041). The prognosis of patients with high SII-PNI score was significantly worse than that of patients with low SII-PNI score (p<0.001). Multivariate analysis showed that SII-PNI score was an independent prognostic factor for predicting overall survival and progression-free survival (p=0.001, 0.002).

**Conclusion:**

Pretreatment SII-PNI score is an important predictor for the efficacy of GC-CY_1_ patients after NIPS paclitaxel combined with Apatinib conversion therapy, which can help to identify high-risk groups and predict prognosis.

## Introduction

Gastric cancer ranks fifth in morbidity and fourth in mortality worldwide (1). Distant metastasis of gastric cancer mainly occurs through blood, lymphatic and direct invasion of adjacent organs (2). However, the most common type of recurrence after treatment in advanced gastric cancer patients is peritoneal metastasis (3, 4). Gastric cancer patients with only positive peritoneal lavage cytology (GC-CY_1_) is defined as the presence of free cancer cells in the abdominal cavity without peritoneal implantation or distant metastasis (5). In recent years, GC-CY_1_ is defined as stage IV in the 15^th^ edition of the Japanese Classification of Gastric Cancer (6). Moreover, the eighth edition of the International Union Against Cancer (AJCC) TNM staging system considers GC-CY_1_ as an independent diagnostic criterion for distant metastasis(M1) (7).

Currently, the prognosis of GC-CY_1_ patients is poor, and there is no universal consensus on the most suitable treatment for these patients (5, 8). Systemic chemotherapy has been widely accepted as the standard treatment for patients with stage IV and has been proved to improve the prognosis (9). However, due to the existence of peritoneal-plasma barrier, chemotherapeutic drugs cannot directly act on the abdominal cavity (10). Therefore, systemic chemotherapy alone is less effective in treating GC-CY_1_ patients. The results of PHOENIX-GC study carried out by Ishigami H eal scholars provided a new treatment idea for GC-CY_1_ patients (11). The combination of systemic chemotherapy and intraperitoneal chemotherapy is considered to be a promising conversion therapy. Meanwhile, Apatinib is an orally active Tyrosine Kinase Inhibitor (TKI), which can effectively inhibit the formation of tumor blood vessels, thus playing an anti-tumor effective and well tolerated for various malignant tumors (12–15). Our previous study found that neoadjuvant intraperitoneal and systemic (NIPS) paclitaxel combined with Apatinib has achieved good results in the conversion treatment of GC-CY_1_ patients, and the R0 resection rate is 77.78% (16). Unfortunately, some patients are still unable to benefit from NIPS paclitaxel combined with Apatinib because of the heterogeneity of gastric cancer or tumor insensitivity to its uniformity (17). However, there is still a lack of reliable indicators to predict efficacy and prognosis of patients before conversion treatment, which might help to optimize the treatment strategies.

Growing evidence show that the occurrence and development of gastric cancer are closely related to the systemic inflammatory response (17, 18). Systemic immune-inflammatory index (SII) is a new inflammatory indicator based on the counts of peripheral blood neutrophils, lymphocytes, and platelets, which can comprehensively reflect the inflammatory response of the body (19). Many studies have confirmed that SII is closely related to the prognosis of various malignant tumors (20, 21). Meanwhile, a study have showed that nutritional status during treatment was also a key factor affecting chemotherapy response (22). As a simple and feasible nutritional detection index, prognostic nutritional index (PNI) is confirmed to be related to the prognosis of various malignant tumors, and is widely used to evaluate the occurrence of perioperative complications and predict the prognosis (23). Previous studies generally used inflammatory markers such as neutrophil-lymphocyte ratio (NLR) and platelet-lymphocyte ratio (PLR) to evaluate the prognosis of patients with gastric cancer (24, 25). However, there are few studies on the efficacy and prognosis of NIPS paclitaxel combined with Apatinib in GC-CY_1_ patients using SII combined with PNI.

In this study, we evaluated the predictive effect of pre-treatment SII-PNI score on efficacy and prognosis in GC-CY_1_ patients after NIPS paclitaxel combined with Apatinib conversion treatment to determine the optimal parameters for predicting survival and clinical response to this combined regimen.

## Materials and Methods

### Study Design and Participants

This is a prospective clinical study of NIPS paclitaxel combined with Apatinib for GC-CY_1_ patients in the Fourth Hospital of Hebei Medical University from April 2018 to August 2019. This trial was registered at Clinical Trials. gov: NCT03718624, and approved by the Ethics Committee of the Fourth Hospital of Hebei Medical University (approval number: 2018088). All patients and/or the legal guardians/surrogates/power of attorneys were informed about the potential adverse effects and signed informed consents.

The following inclusion criteria were applied: (I) gastric adenocarcinoma confirmed by histopathology and free cancer cells (FCCs) positivity confirmed by exfoliated cells in the abdominal cavity; (II) preoperative computed tomography (CT) imaging showed no distant organ metastasis and no distant lymph node metastasis above the third station; (III) patients aged ≤75 years; (IV) the Eastern Cooperative Oncology Group (ECOG) activity status score was ≤2 points; (V) patients had good bone marrow function(before treatment in patients with peripheral blood examination if there is no bone marrow suppression, or bone biopsy exclude blood system diseases show good bone marrow function), liver function(the peripheral blood test showed that ALT, AST ≤ 2.5*ULN and TBIL< 1.5*ULN), heart function(no atrial fibrillation, angina pectoris, cardiac insufficiency, ejection fraction less than 50% and poor hypertension control), and kidney function(the peripheral blood test showed that serum creatinine ≤ 1.5*ULN before treatment), and were able to tolerate chemotherapy; (VI) there were no other serious immunosuppressive diseases or simultaneous malignant tumors; (VII) and pathological human epidermal growth factor receptor 2 (HER2) tests were negative prior to the operation. Patients were excluded if they presented with the following: (I) difficulty taking oral medications (such as dysphagia, chronic diarrhea, and gastrointestinal obstruction, etc.); (II) high blood pressure that could not be controlled by a single antihypertensive drug treatment; (III) 24 hour urine protein quantification >1.0 g; (IV) imaging results showing the tumor had invaded important blood vessels or the investigator judged that the tumor was highly likely to invade important blood vessels during treatment and cause fatal bleeding; (V) abnormal blood coagulation; and (VI) other comorbidities that may seriously endanger the safety of the patient or affect the completion of the study as determined by the investigator.

### Chemotherapy Regimen

The treatment regimens of all patients in this study were consistent with our previous study (16). Treatment commenced on the day after the laparoscopic exploration, and each cycle of treatment lasted for 3 weeks. On the 1st and 8th day of the treatment cycle, paclitaxel was infused *via* an intraperitoneal (IP) chemotherapy pump (IP route 20 mg/m^2^, dissolved in 1,000 mL of normal saline, infusion for more than 1 hour) and intravenously (IV) (IV route 50 mg/m^2^, dissolved in 500 mL of saline, infusion for more than 1 hour). Dexamethasone and cimetidine were administered before paclitaxel treatment. Oral S-1 (a contemporary oral fluoropyrimidine) 80 mg/(m^2^·d) was given 30 minutes after breakfast and 30 minutes after dinner for 14 consecutive days. At the same time, Apatinib 500 mg/d was administered orally for 21 consecutive days. The dose of S-1 was determined according to the body surface area (BSA) as follows: for BSA <1.25 m^2^, 80 mg/(m^2^·d) S-1 was administered; for BSA 1.25-1.50 m^2^, 100 mg/(m^2^·d) S-1 was administered; and for BSA >1.50 m^2^, 120 mg/(m^2^·d) S-1 was given. After one month of rest, radical D2 operation was arranged, and then another six cycles of NIPS paclitaxel combined with Apatinib conversion treatment were repeated 1 month postoperatively.

### Assessments

Four weeks after the completion of three cycles of NIPS paclitaxel combined with Apatinib conversion therapy, the objective efficiency and resectability of the tumor were evaluated by computed tomography(CT). Tumor response was assessed based on the rules established by the Response Evaluation Criteria in Solid Tumors (RECIST) 1.1 (3), which was divided into complete response (CR), partial response (PR), stable disease (SD) and progressive disease (PD).

And laparoscopic exploration was performed again. If free cancer cells (FCCs) negative confirmed by exfoliated cells in the abdominal cavity and are defined as non-FCCs, then standard D2 lymph node dissection is performed. However, if FCCs were still detected in the abdominal cavity, then the original chemotherapy regimen would be continued. And in this study, all patients were divided into FCCs group and non-FCCs group according to FCCs status after NIPS paclitaxel combined with Apatinib conversion treatment by re-laparoscopy and peritoneal cytology.

### Definitions and Follow-up

The peripheral venous blood was collected in fasting state within one week before chemotherapy in all patients. The counts of peripheral neutrophils, lymphocytes, and platelets were measured and analyzed by an automatic blood analyzer (Beckman Coulter LH750), and the levels of peripheral albumin were measured and analyzed by an automatic blood analyzer (Beckman Coulter AU5800). The definitions of PNI and SII were shown as follows: PNI= albumin (g/L) + 5×total lymphocyte counts(10^9^/L) (26); SII= platelet × neutrophil/lymphocyte counts (27).

All patients were recommended to have a follow-up visit every 3 months in the first 2 years, and every 6 months after 2 years. Follow-up methods mainly include telephone encounter, outpatient visits, and hospitalization. The hospital examination items included CT of chest, abdomen, and pelvis, as well as esophagogastroduodenoscopy (EGD) and tumor markers. In this study, the deadline for follow-up was September, 1^st^, 2021. Overall survival (OS) was defined as the time interval from treatment to cancer-related death or final contact, and OS was the preferred destination. And progression-free survival (PFS) was measured from the time of treatment initiation to clinical or radiographic progression or death from any cause.

### Statistical Analyses

SPSS version 21.0 and GraphPad Prism 5.01 were used for statistical analyses. The receiver operating characteristic curve (ROC) and the area under the ROC Curve (AUC) was used to evaluate the predictive ability of SII and PNI in distinguishing FCCs patients and non-FCCs patients, and the optimal cut-off values of SII and PNI with the highest Youden index were obtained. Survival analysis was performed using Kaplan-Meier method. Cox proportional hazards regression model was used for univariate and multivariate analysis. Relative risk was assessed using hazard ratio (HR) and 95% confidence interval (CI). Spearman correlation analysis was used to evaluate the relationship between PNI and SII. p< 0.05 indicated that the difference was statistically significant.

## Results

### Patients’ Demographic Information and Tumor Characteristics

**Figure 1:**
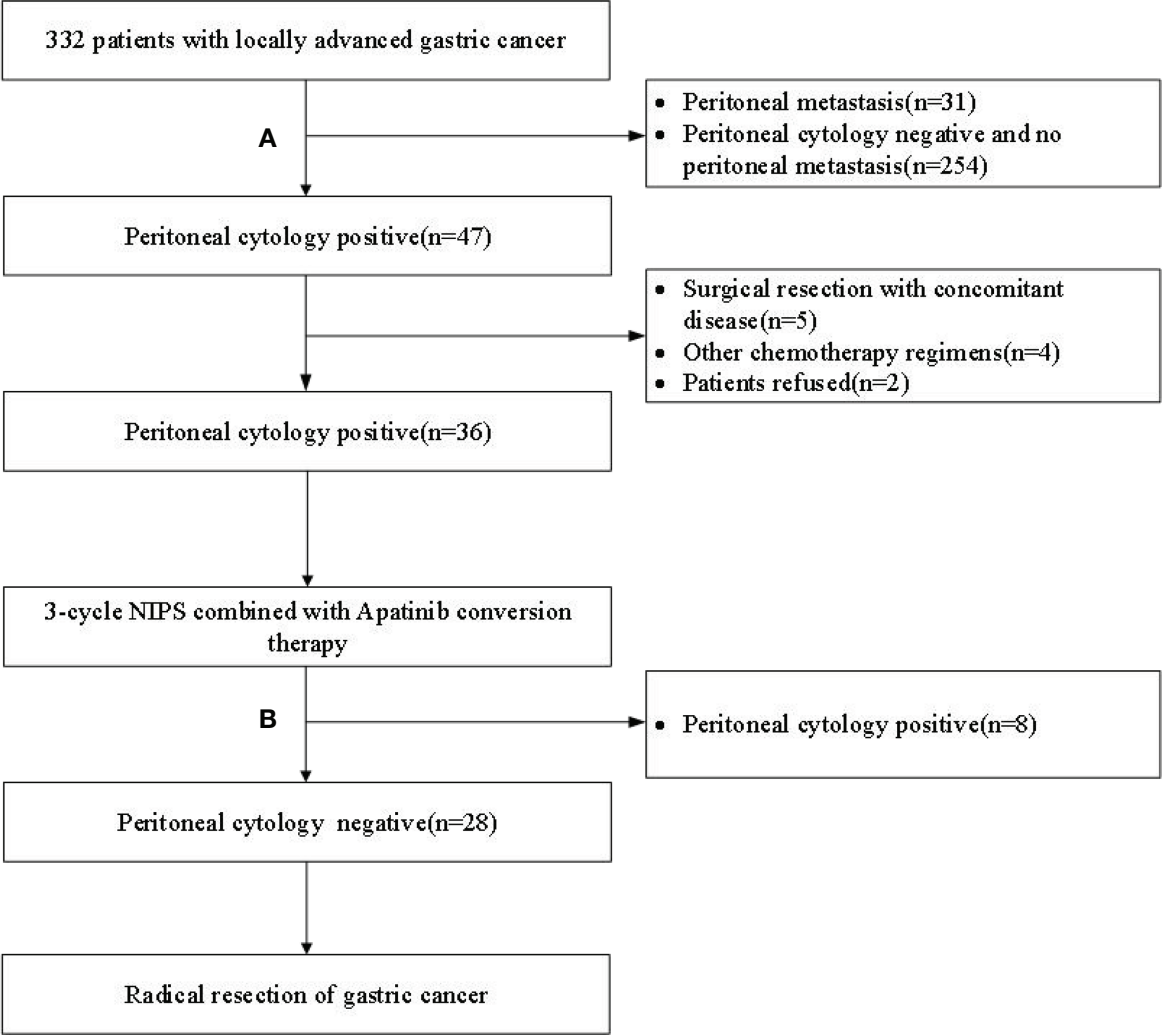
Flow chart of patient enrollment and exclusion. **(A)** Laparoscopic exploration + peritoneal cytology; **(B)** Re-laparoscopic exploration + peritoneal cytology; NIPS, Neoadjuvant intraperitoneal and systemic.

**Figure 2:**
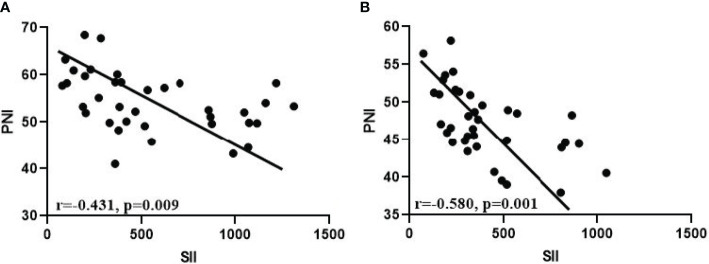
Correlation analysis between SII and PNI. **(A)** Before conversion therapy; **(B)** After 3 cycles of conversion therapy.

This study prospectively included 36 GC-CY_1_ patients according to the inclusion and exclusion criteria (**Figure 1**). The demographic information and tumor characteristics of the patients are summarized in **Table 1**. There were 25 males (69.44%) and 11 females (30.56%). The median age of the patient was 54 years old, ranging from 32 to 66. The tumor lesions was ≥5 cm in diameter (75.00%) in 27 cases, and less than 5 cm in 9 cases (25.00%). The median values of pre-treatment SII and PNI were 328.4 and 53.3, respectively, while the median values after three cycles of conversion treatment were 328.4 and 46.9, respectively. Meanwhile, before conversion therapy(r=-0.431, p*=*0.009; **Figure 2A**) and after 3 cycles of conversion therapy(r=-0.580, p*=*0.001; **Figure 2B**), there is a close negative correlation between the two systemic indicators of SII and PNI.

**Table 1:** Patient demographic information and tumor characteristics.

Characteristics	Case (%)	Mean (SD)	Range
**Sex**			
Male	25 (69.44)		
Female	11 (30.56)		
**Age (years)**		54 (10.4)	32-66
**ECOG performance status**			
0	30 (83.33)		
1	6 (16.67)		
**Tumor size (cm)**		7.8 (3.2)	3.4-10.6
<5.0	9 (25.00)		
≥5.0	27 (75.00)		
**Differentiation**			
Poor	30 (83.33)		
Moderately or well	6 (16.67)		
**Lesion site**			
Cardia	13 (36.11)		
Stomach	4 (11.11)		
Gastric antrum	14 (38.89)		
Whole stomach	5 (13.89)		
**Pathological T stage**			
T3	6 (16.67)		
T4	30 (83.33)		
**Pre-treatment SII**		553.6 (372.5)	77.5-1311.2
**Pre-treatment PNI**		54.3 (6.3)	41.0-68.5
**Posttreatment SII**		402.0 (247.5)	72.6-1048.0
**Posttreatment PNI**		47.3 (4.8)	38.0-58.2

ECOG, Eastern Cooperative Oncology Group; SII, Systemic immune-inflammatory index; PNI, prognostic nutritional index.

### Optimal Cut-Off Values of SII and PNI Before and After Conversion Treatment

36 GC-CY_1_ patients underwent laparoscopic exploration combined with peritoneal cytology after 3 cycles of NIPS paclitaxel and Apatinib conversion therapy. Among them, 28 cases (77.78%) were FCCs negative (non-FCCs), all negative patients underwent R0 resection. The remaining 8 patients (22.22%) were found to have free cancer cells (FCCs) in the abdominal cavity, and continued the original chemotherapy regimen of Apatinib conversion therapy. After 3 cycles of conversion therapy, 3 patients were evaluated by CT for local lesion progression, and 5 patients underwent laparoscopic exploration and peritoneal cytology again. Of the 5 patients, only 1 was negative, 2 were still positive and 2 had peritoneal metastasis.

**Figure 3:**
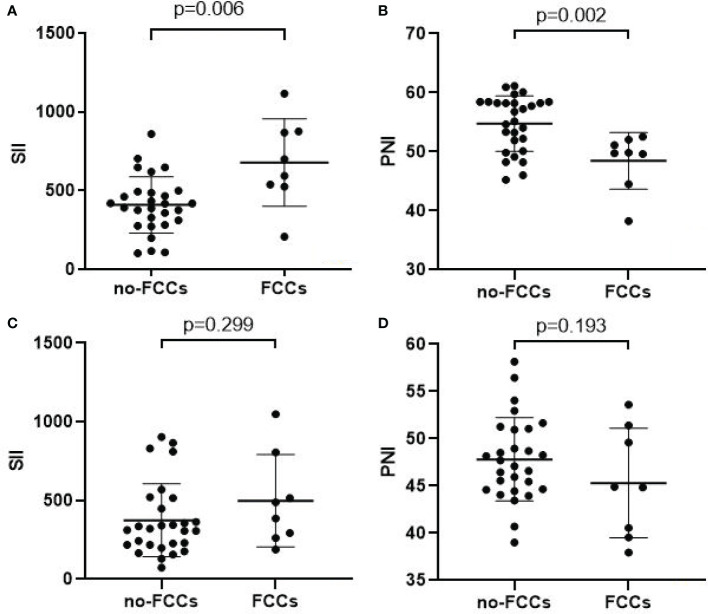
Relationship between tumor response and the SII(A/C)/PNI(B/D). **(A, B)** Before conversion therapy; **(C, D)** After 3 cycles of conversion therapy.

The mean SII and PNI in the 28 patients with non-FCCs were 408.9 ± 179.1 and 54.8 ± 4.7, respectively. Meanwhile, the mean pre-treatment SII and PNI in the 8 patients with FCCs were 677.8 ± 277.6 and 48.5 ± 4.8, respectively. The pre-treatment SII in FCCs patients was significantly higher than that in non-FCCs patients (p=0.006) (**Figure 3A**), while the PNI in FCCs patients was lower than that in non-FCCs patients (p=0.002) (**Figure 3B**). Furthermore, we found that after three cycles of conversion therapy, the average levels of SII and PNI in 28 patients with no-FCCs were not significantly different from those in 8 patients with FCCs (374.5 vs. 498.3, p=0.299; 47.8 vs. 45.3, p=0.193) (**Figures 3C, D**).

**Figure 4:**
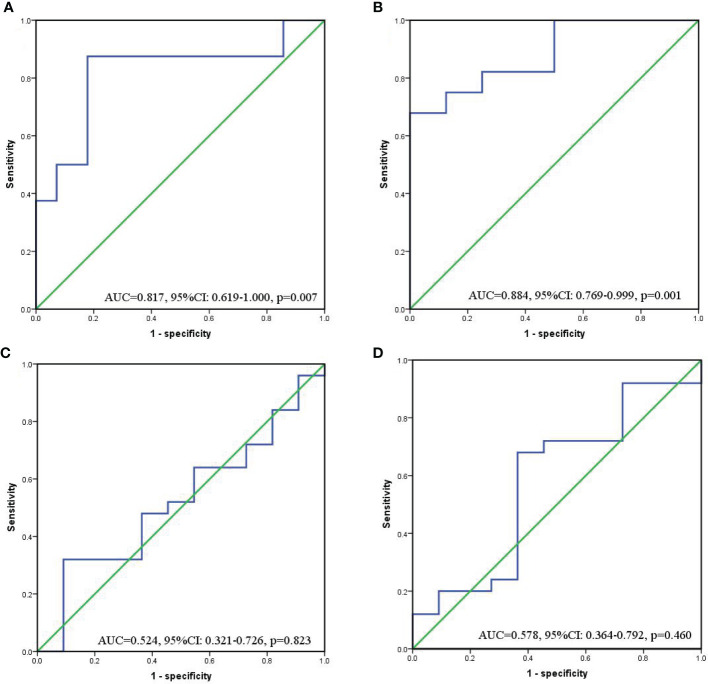
ROC curves for discriminating patients with FCCs and those with non-FCCs according to values of the SII **(a/c)** and PNI **(b/d)**. **(A, B)** Before conversion therapy; **(C, D)** After 3 cycles of conversion therapy.

In order to determine the cut-off value of continuous variables, we constructed ROC curves and calculated AUC to evaluate the changes of SII and PNI before and after conversion therapy to distinguish FCCs and non-FCCs patients. The optimal cut-off value of SII before conversion therapy was 512.1 (AUC=0.817, 95%CI: 0.619-1.000, p=0.007), and the corresponding sensitivity was 0.875 and specificity was 0.821 (**Figure 4A**). The optimal cut-off value of PNI was 52.9 (AUC=0.884, 95%CI: 0.769-0.999, p=0.001), with the corresponding sensitivity of 0.679 and specificity of 0.863 (**Figure 4B**). However, after the three-cycle translational therapy, the optimal cut-off value of SII was 487.5 (AUC=0.524, 95%CI: 0.321-0.726, p=0.823), and the optimal cutoff value of PNI was 46.9 (AUC=0.578, 95%CI: 0.364-0.792, p=0.460), which failed to accurately distinguish FCCs and non-FCCCs patients (**Figures 4C, D**). According to the optimal cut-off values of SII and PNI before conversion therapy, all patients were divided into three group: score of 2 (n=10), high SII (≥512.1) and low PNI (≤52.9); score of 1(n=13), either high SII or low PNI; score of 0(n=13), no high SII nor low PNI.

### The Relationship Between SII-PNI Score and Curative Effect of Conversion Therapy

**Figure 5:**
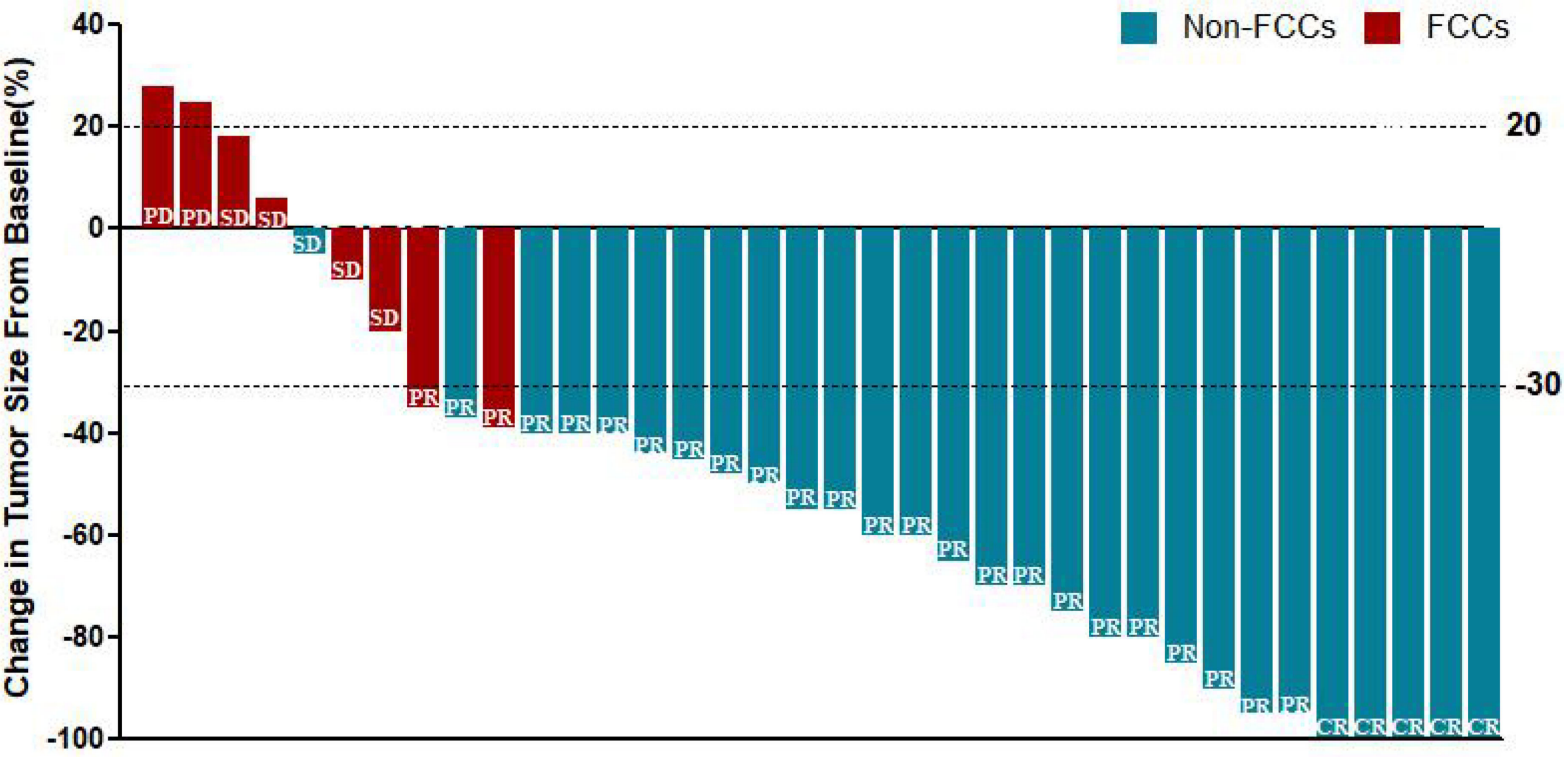
A waterfall plot of ranked best tumor shrinkage.

All patients received 3 cycles of NIPS paclitaxel combined with Apatinib conversion therapy and the whole abdominal enhanced CT scan was evaluated by RECIST 1.1. According to RECIST criteria, there were 5 cases of CR (13.89%), 24 cases of PR (66.67%), 5 cases of SD (13.89%), and 2 cases of PD (5.56%) (**Figure 5**). There was no difference in SII-PNI score between non-PD patients and PD patients (p=0.534) (**Table 2**). However, the SII-PNI score was significantly lower in patients with non-FCCs than in those with FCCs (p=0.041) (**Table 3**).

**Table 2:** Relationship between tumor response and the SII-PNI score.

Tumor response	SII-PNI score(%)			p value
	0 (n=13)	1 (n=13)	2 (n=10)	
Non-PD (n=34)	13 (100)	12 (92.31)	9 (90.00)	0.534
PD (n=2)	0 (0)	1 (7.69)	1(10.00)	

**Table 3:** Relationship between FCCs status and the SII-PNI score.

FCCs status	SII-PNI score(%)			p value
	0 (n=13)	1 (n=13)	2 (n=10)	
Non-FCCs (n=28)	12 (92.31)	11 (84.62)	5 (50.00)	0.041
FCCs (n=8)	1 (7.69)	2 (15.38)	5 (50.00)	

### Relationship Between SII-PNI Score and Prognosis

**Figure 6:**
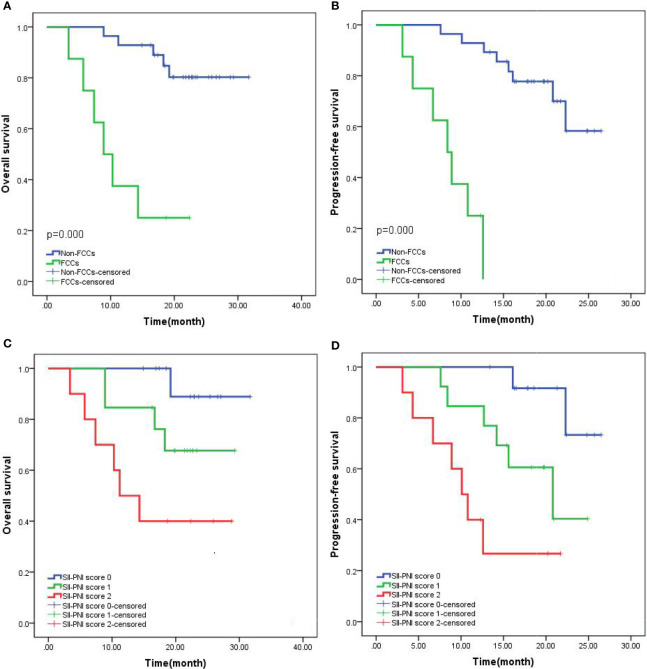
Kaplan-Meier survival curves in patients with GC-CY_1_. **(A, B)** 2-year overall survival, progression-free survival based on FCCs status; **(C, D)** 2-year overall survival, progression-free survival based on SII-PNI score.

All patients were followed up with the median follow-up period of 25.5 months (15.6-38.4months). The 2-year OS was 69.44% and the median overall survival (mOS) was 19.9 months (95%CI: 6.9-31.7 months). The 2-year progression-free survival (PFS) was 58.33%, and the median PFS (mPFS) was 17.2 months (95%CI: 5.9-26.5 months). Subgroup analysis showed that the 2-year OS (82.14% vs 25.00%, p=0.000) and PFS (71.43% vs 12.50%, p=0.000) of non-FCCs group were better than those of FCCs group after re-laparoscopic exploration (**Figures 6A, B**). Meanwhile, the 2-year OS of patients with SII-PNI score of 0, 1, and 2 were 92.31%, 69.23%, and 40.00%, respectively, and the difference between the three groups was significant (all p<0.001, **Figure 6C**). And, the 2-year PFS of the three groups was 84.62%, 53.85%, and 30.00%, respectively, and the difference was significant among the three groups (all p<0.001, **Figure 6D**). Multivariate analysis showed that SII-PNI score (p=0.001, p=0.002), tumor differentiation (p=0.031, p=0.029), and the FCCs status after NIPS paclitaxel combined with Apatinib conversion therapy (p=0.001, p=0.003) were all independent risk factors affecting 2-year OS and PFS of GC-CY_1_ patients (**Tables 4**).

**Table 4:** Multivariate analysis of the clinicopathological characteristics for the prognosis of GC-CY_1_ patients.

Independent factor	2-year OS Multivariate analysis			2-year PFS Multivariate analysis		
	Hazard ratio	95% CI	p value	Hazard ratio	95% CI	p value
Sex			0.315			0.143
Female	1.000	reference		1.000	reference	
Male	1.066	0.521-1.412		1.243	0.721-1.772	
Age (years)			0.083			0.251
≤50	1.000	reference		1.000	reference	
>50	1.268	0.897-1.879		1.153	0.790-1.553	
FCCs status			0.001			0.003
Non-FCCs	1.000	reference		1.000	reference	
FCCs	5.578	3.426-10.142		5.114	3.234-9.981	
SII-PNI score			0.001			0.002
0	1.000	reference		1.000	reference	
1	1.748	1.541-3.632		1.927	1.077-2.774	
2	3.576	2.578-6.895		3.152	1.569-5.072	
Tumor size (cm)			0.052			0.061
<5.0	1.000	reference		1.000	reference	
≥5.0	1.920	1.256-3.492		1.759	1.352-3.152	
Differentiation			0.031			0.029
Poor	1.000	reference		1.000	reference	
Well	2.571	1.287-3.379		2.496	1.772-4.218	

SII, Systemic immune-inflammatory index; PNI, Prognostic nutritional index; FCCs, Free cancer cells.

## Discussion

At present, it is believed that the positive peritoneal FCCs are the early stage of peritoneal colonization in gastric cancer, which is called GC-CY_1_ (3). These patients have poor prognosis and poor surgical treatment effect, and the median survival time is 12 months (28). There are differences in the treatment strategies for GC-CY_1_ patients worldwide. The fifth edition of the Guidelines for the Treatment of Japanese Gastric Cancer Association proposed that if GC-CY_1_ patients did not have other distant organ metastasis, they could receive surgical treatment at first and postoperative chemotherapy to further prolong survival (29). However, the National Comprehensive Cancer Network (NCCN) guidelines in the United States recommend that GC-CY_1_ patients should be treated according to the principle of advanced gastric cancer (30). Chemotherapy should be carried out first, and then exploration can be carried out again after chemotherapy. Patients with negative intraperitoneal FCCs may benefit from surgical treatment, while patients with persistent positive FCCs are recommended to continue chemotherapy (3). Meanwhile, our previous study has confirmed that NIPS paclitaxel combined with Apatinib was effective in the treatment of GC-CY_1_ patients and prolonged their survival time (16). Nevertheless, not all patients can benefit from it, with about 22% of the disease progression after treatment. For these patients, this combination treatment not only increases the relevant medical costs, but also may weaken the immune system and delay the best timing of surgery. Therefore, before NIPS paclitaxel combined with Apatinib is carried out for patients with GC-CY_1_, a simple indicator to accurately predict the therapeutic effect will be beneficial to the formulation and selection of individualized treatment regimens.

In recent years, an increasing number of studies have confirmed that inflammatory response is closely related to the occurrence and development of tumors (31, 32). As a systemic inflammatory response indicator, SII has been confirmed to be closely related to the prognosis of patients with gastric cancer by many studies, which can be used to predict the prognosis of patients (19, 33). On the other hand, numerous studies have shown that malnutrition not only affects the clinical decision-making of cancer treatment, but also increases the incidence of complications and mortality, and reduces the quality of life of patients (34, 35). As an indicator reflecting the nutritional status of patients, PNI is widely used to evaluate the occurrence of perioperative complications and predict the prognosis (36, 37). To the best of our knowledge, we were the first to combine the SII and PNI of GC-CY_1_ patients before receiving NIPS paclitaxel combined with Apatinib conversion therapy to establish the SII-PNI score as a new scoring system for predicting the efficacy and prognosis of patients.

Previous studies have shown that FCCs status is one of the most important factors to evaluate the effectiveness of GC-CY_1_ patients after conversion therapy (5). Unfortunately, the curative effect of treatment is difficult to predict by using clinical pathological information before conversion treatment. Therefore, we focus on pre-treatment SII and PNI to overcome the challenges associated with predicting therapeutic efficacy. Previous studies have shown that SII can be used to predict the pathological complete remission and prognosis of patients after neoadjuvant chemotherapy for breast cancer (38, 39). PNI is also widely used to evaluate the efficacy and prognosis of chemotherapy for advanced non-small cell lung cancer and colorectal cancer (40, 41). However, the application of systemic inflammatory response index combined with nutritional status index, namely the SII-PNI scoring system to predict the efficacy and prognosis of GC-CY_1_ patients receiving NIPS paclitaxel and Apatinib conversion therapy is rarely reported. This study analyzed the relationship between SII-PNI score and efficacy of NIPS paclitaxel combined with Apatinib in GC-CY_1_ patients after conversion therapy. The results of this study showed that the SII-PNI score before treatment was closely related to the efficacy of conversion therapy. The lower SII-PNI score before treatment, the more likely the FCCs positive will turn to negative, and the more likely the NIPS paclitaxel combined with Apatinib conversion treatment will be successful. This suggests that the SII-PNI score may be a promising candidate for predicating the efficacy response of GC-CY_1_ patients after receiving NIPS paclitaxel combined with Apatinib conversion therapy.

We also evaluated the relationship between SII-PNI score and prognosis. The 2-year OS of patients with SII-PNI score of 0, 1, and 2 were 92.31%, 69.23%, and 40.00%, respectively, and the difference between the three groups was significant. Meanwhile, similar results were also obtained in patients with PFS. Furthermore, this study analyzed the risk factors that may affect the survival of GC-CY_1_ patients receiving NIPS paclitaxel combined with Apatinib conversion treatment, and found that the SII-PNI score was an independent risk factor affecting the 2-year OS and PFS of patients. The possible mechanism of SII-PNI predicting prognosis are as the followings: The higher SII-PNI score indicates a relative increase of neutrophil and/or platelet counts. Neutrophils release active nitrogen, reactive oxygen species, and elastase, activate the P13K-AKT signaling pathway, and promote the proliferation of tumor cells (36, 42). In addition, platelets may play a certain role in the growth, proliferation, and metastasis of tumors, mainly by secreting related tumor growth factors to promote tumor growth. Platelets are also involved in the escape of tumor cells from the host immune system (43). The increase in SII-PNI score also indicates that lymphocytes are relatively reduced, leading to a reduced immune regulation function and promoting the progression of tumor deterioration (44). The decrease of serum albumin level in the body reflects the decrease of nutritional status of patients (45). The worse the nutritional status is, the lower the immunity of the body will be, which will lead to the progress of disease.

It is noteworthy that a few limitations of current research also exist. First, this prospective study was conducted in a single center with a small sample size (n=36), which is the main limitation. Second, this study only selected NIPS paclitaxel combined with Apatinib for analysis. Therefore, larger, multi-center prospective studies investigating different treatment regimens are urgently needed to confirm our findings.

## Conclusions

In conclusion, our study suggests that the SII-PNI score is a promising predictor of the efficacy and survival outcomes of GC-CY_1_ patients after NIPS paclitaxel combined with Apatinib conversion therapy. These findings may be beneficial to the formulation of therapeutic strategies and clinical risk stratification to avoid unnecessary toxicity/adverse effects in patients who are unlikely to benefit from treatment.

## Data Availability Statement

The raw data supporting the conclusions of this article will be made available by the authors, without undue reservation.

## Ethics Statement

The studies involving human participants were reviewed and approved by Ethics Committee of the Fourth Hospital of Hebei Medical University (approval number: 2018088). The patients/participants provided their written informed consent to participate in this study.

## Author Contributions

Contributions: (I) Conception and design: QZ. (II) Administrative support: QZ. (III) Provision of study materials or patients: P’aD, PY, YT, HG, and YaL. (IV) Collection and assembly of data: P’aD, PY, YT, and HG. (V) Data analysis and interpretation: P’aD and CS. (VI) Manuscript writing: All authors. All authors contributed to the article and approved the submitted version.

## Funding

This work was supported by the Cultivating Outstanding Talents Project of Hebei Provincial Government Fund (No.2019012); Hebei public health committee county-level public hospitals suitable health technology promotion and storage project (No.2019024); Hebei Medical University Education and Teaching Research Project (No.2020CGPY-12, No.2020CHYB-23); Hebei University Science and Technology Research Project (No.ZD2019139).

## Conflict of Interest

The authors declare that the research was conducted in the absence of any commercial or financial relationships that could be construed as a potential conflict of interest.

## Publisher’s Note

All claims expressed in this article are solely those of the authors and do not necessarily represent those of their affiliated organizations, or those of the publisher, the editors and the reviewers. Any product that may be evaluated in this article, or claim that may be made by its manufacturer, is not guaranteed or endorsed by the publisher.
